# Responses to “Bio-optimized *Curcuma longa* extract is efficient on knee osteoarthritis pain: a double-blind multicenter randomized placebo controlled three-arm study”: authors’ reply

**DOI:** 10.1186/s13075-020-2109-2

**Published:** 2020-02-11

**Authors:** Yves Henrotin, Anne-Françoise Donneau, Kurt de Vlam, Ruth Wittoek, Frank Luyten

**Affiliations:** 1 Bone and Cartilage Research Unit, Institute of Pathology, Level 5, University of Liège, Liège, Belgium; 2 Public health Science Department, University of Liège, Liège, Belgium; 3 Rheumatology Department, ZNA Jan Palfijn, Merksem, Belgium; 4 Rheumatology Department, UZ Gent, Ghent, Belgium; 5 Rheumatology Department, University Hospitals Leuven, Leuven, Belgium

The paper published by Henrotin et al. [1] reported the results from a double-blind multi-center randomized placebo controlled study of bio-optimized *Curcuma longa* (BCL) extract in patients with symptomatic knee osteoarthritis (OA). This bio-optimized curcuma extract is a widely used food supplement.

We received two letters that highlight six potential shortcomings in the design of this study and the interpretation of the results. As the content of these letters is virtually similar, we decided to answer both letters in one document.

The first shortcoming was about the timeline of study registration. This prospective study was recorded in September 2016 while the first patient was enrolled in 2014. In contrast with medicinal products and biologics and medical devices, there are no legal requirements for registry of a clinical trial investigating dietary supplements. However, in order to comply with the Declaration of Helsinki and WHO recommendations, for the sake of transparency, and for scientific publication, the trial was indeed registered on Clinical trial.gov on September 13, 2016 (NCT02909621).

The second point concerns the selection of the co-primary endpoints. A particularity of this study was the use of two unusual co-primary endpoints. One endpoint was the Patient Global Assessment of Disease Activity (PGADA) and the other the serum levels of Coll2-1, a specific peptide and epitope of type II collagen and considered as a biomarker of cartilage degradation. Indeed, these co-primary endpoints are not recommended by any regulatory agency, but they were defined based on the results obtained in a previous pilot study [2]. Importantly, the design of this study was exploratory and gave interesting data about innovative biomarkers. Further, this approach was in line with OARSI-FDA consortium recommendation 23 which encouraged the collection of biological fluids in randomized clinical trials to investigate the metabolic effect of a treatment on joint tissues and to qualify a biomarker or a cluster of biomarkers for treatment development and use in the context of a personalized approach of OA management [3]. Again, this study has an objective to provide useful information for the design of a potential larger phase III study including the sample size estimate, the choice of the dose, and the selection of primary outcome.

The third point concerns the calculation of the sample size. The sample size calculation was challenged by the statistician of the journal and ultimately considered as correctly addressed. The sample size was calculated following the highest standard. Indeed, the sample size was defined according the International Clinical Harmonization (ICH) E9 guidelines for clinical trials (ICH E9—see additional file 2 in the original paper). Sample size considered also the results obtained in a preceding pilot study [2]. We calculated a sample size of 150 patients based on a decrease in Coll2-1 biomarker levels of 44.4 nmol/L with standard deviation of 53 or a decrease in patient assessment of global disease activity of 21 mm with standard deviation of 25 in the treated groups and no change in the control group, an alpha risk of 5% that was divided by 2 (2.5%) as a correction for multiple testing, a power of 85%, and a drop-out rate of 15%. Taken individually, the two co-primary endpoints lead to the same sample size.

The fourth comment questioned the role of the 6-month test results. As explained in the original paper, the 6-month follow-up was exploratory and failed to give additional relevant information. For that reason, the 6-month data were not reported in the paper but presented in supplementary tables.

Fifth, only post hoc analyses reported a difference compared to the placebo group, which is methodologically disputable in a study that fails to meet the primary endpoints. Post hoc analysis is commonly performed in the literature, even in a negative study, and cannot be considered as methodologically disputable. Of course, results have to be interpreted and discussed in consideration of this point.

The final shortcoming was about the safety of the product. As indicated in the original paper, 18.5% of the subjects dropped out of the trial because of adverse events in the high dose group. Consequently, the authors of the study suggested that the recommended dose of BCL was two caps twice a day. This observation met one the objectives of the study, which was to identify the potentially most effective and safe dose of BCL.

In conclusion, we would like to point out that this study was performed according to the International Council for Harmonisation of Technical Requirements for Pharmaceuticals for Human use (ICH E6) and interpretation consistent with the results, balancing benefits and harms, and considering other relevant evidence.

We believe that the paper conclusion “efficacy analysis suggested positive trends for measurements of PGADA and serum levels of a biomarker and a significant decrease of pain in knee OA” is reasonably well balanced. It has not escaped our attention that the authors of these letters fail to disclose their potential conflicts of interest.

## Data Availability

All the data are available and can be requested at Tilman SA (Baillonville, Belgium, yd@tilman.be).

## Abbreviations

BCL: Bio-optimized *Curcuma longa*
ICH: International Clinical Harmonization
OA: Osteoarthritis
PGADA: Patient Global Assessment of Disease Activity

## References

[1] Henrotin Y, Malaise M, Wittoek R (2019). Bio-optimized Curcuma longa extract is efficient on knee osteoarthritis pain: a double-blind multicenter randomized placebo controlled three-arm study. Arthritis Res Ther.

[2] Henrotin Y, Gharbi M, Dierckxsens Y (2014). Decrease of a specific biomarker of collagen degradation in osteoarthritis, Coll2-1, by treatment with highly bioavailable curcumin during an exploratory clinical trial. BMC Complement Altern Med.

[3] McAlindon TE, Driban JB, Henrotin Y, Hunter DJ, Jiang GL, Skou ST, Wang S, Schnitzer T (2015). OARSI Clinical Trials Recommendations: design, conduct, and reporting of clinical trials for knee osteoarthritis. Osteoarthr Cartil.

